# Ten years’ disease-free survival of advanced epithelial ovarian cancer treated by cytoreductive surgery plus hyperthermic intraperitoneal chemotherapy: A case report and literature review

**DOI:** 10.1097/MD.0000000000023404

**Published:** 2020-12-04

**Authors:** Jue Zhang, Liejun Mei, Fubing Wang, Yan Li

**Affiliations:** a Department of Peritoneal Cancer Surgery, Beijing Shijitan Hospital, Capital Medical University, Beijing 100038, PR. China; b Department of Radiology, Zhongnan Hospital of Wuhan University, Wuhan 430071, PR. China; c Department of Clinical Laboratory, Zhongnan Hospital of Wuhan University, Wuhan 430071, PR. China.

**Keywords:** advanced ovarian cancer, cytoreductive surgery, hyperthermic intraperitoneal chemotherapy, peritoneal carcinomatosis

## Abstract

**Rationale:**

One of the most distinctive features of epithelial ovarian cancer is tendency to disseminate into peritoneal cavity to form peritoneal carcinomatosis, indicating advanced disease with poor prognosis.

**Patient concerns:**

A fifty-year-old patient had a chief complaint of an abdominal distension lasting 1 month.

**Diagnoses:**

The patient was diagnosed with advanced epithelial ovarian cancer with peritoneal carcinomatosis by computed tomography scan, tumor markers, endoscopy examination, and pathology.

**Interventions:**

The patient was treated with cytoreductive surgery plus hyperthermic intraperitoneal chemotherapy followed by 8 cycles of systemic chemotherapy.

**Outcomes:**

Till March 9, 2020, the patient has disease-free survival over 10 years.

**Lessons:**

The application of cytoreductive surgery plus hyperthermic intraperitoneal chemotherapy combined with systematic chemotherapy may improve survival dramatically for the patients with epithelial ovarian cancer and peritoneal carcinomatosis and should be considered as an option of a relatively new regime.

## 1 Introduction

Epithelial ovarian cancer (EOC) is one of the main causes of cancer death for women worldwide.^[1]^ It is often diagnosed at an advanced stage and the disease remains confined to the peritoneal carcinomatosis (PC) for much of its natural history, resulting in unfavorable outcomes, with an overall 5-year survival rate <20%.^[2]^ Since 1980s, the standard care for EOC-PC has been undertake debulking surgery followed by systemic chemotherapy with platinum and taxanes-based regimens.^[3]^

Over the past 30 years, cytoreductive surgery (CRS) plus hyperthermic intraperitoneal chemotherapy (HIPEC) has been developed to treat PC from various abdominal and pelvic malignancies, which combines the advantages of CRS to remove the visible tumor nodules, and HIPEC to eradicate micrometastases and free cancer cells. The success of such integrated approach has been verified in PC from colorectal cancer and pseudomyxoma peritonei from appendiceal original. And in recent years, researchers have also applied CRS + HIPEC to treat EOC-PC,^[4]^ with encouraging results. In particular, 3 prospective randomized controlled trials organized by the Gynecologic Oncology Group (GOG) have shown that CRS + HIPEC could improve the overall survival (OS) of patients with selected EOC-PC.^[3,5,6]^ The sound clinical evidence prompts the National Cancer Institute (ASCO, American Society of Clinical Oncology) to recommend combined intravenous and intraperitoneal chemotherapy for stage III ovarian cancer. NCCN has recommended intraperitoneal chemotherapy as an optimal treatment option for ovarian cancer.

In China, our group has been focused on CRS + HIPEC to treat PC from gastrointestinal and gynecological cancers. And in this report, we present a successful case of EOC-PC treated by CRS+HIPEC, who has remained disease-free for over 10 years. Pertinent literatures are also reviewed.

## 2 Case presentation

**Figure 1:**
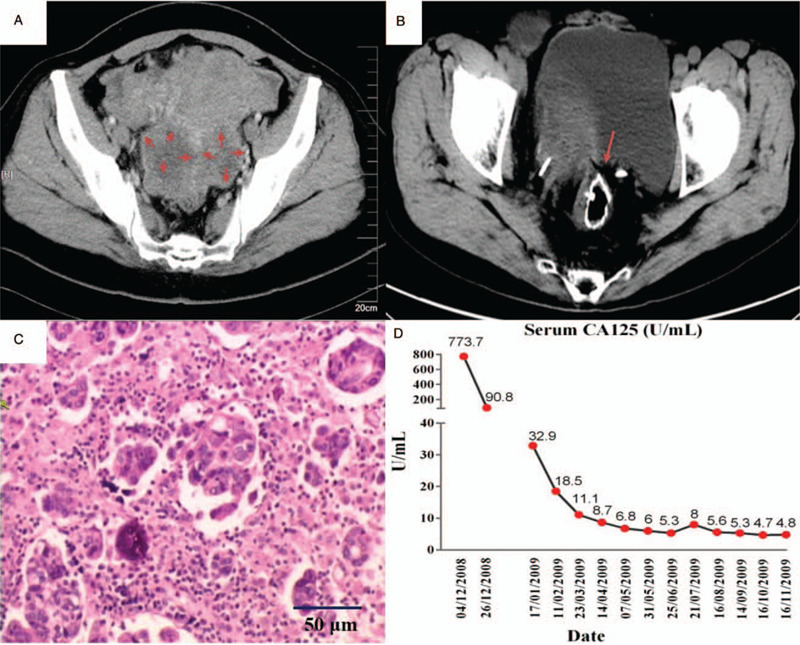
Related results of the patient's treatment process. (A) Before the first surgery, contrast-enhanced CT scan shows huge pelvic malignant tumors, with adhesion to the rectum and sigmoid colon. (B) After the second surgery, abdominopelvic CT scan after CRS + HIPEC shows a clean and clear pelvic floor, with circular anastomosis marker of descending colon and rectum (the red arrow). (C) Pathological analysis on the tumors. (Bilateral) ovarian serous papillary adenocarcinoma (grade II-III). HE staining (×400). (D) Serum CA125 levels during the treatment course, which remain normal range after CRS+HIPEC and consolidation chemotherapy.

A 50-year-old female patient was referred to our clinic on November 11, 2008, complaining about an abdominal distension that lasted one month. The patient had hypertension and nephrolith and did not have family history of cancer. Gastrointestinal endoscopy and colonoscopy identified no neoplastic lesion. Abdominopelvic doppler ultrasonography and contrast-enhanced computed tomography (CT) scan revealed solid pelvic tumors and ascites (Fig. 1A). Carbohydrate antigen (CA) 125 was 773.7 U/mL (normal range 0–35 U/mL), but the other tumor markers, such as CA-199, CA-153, alpha fetoprotein, and routine blood tests had no obvious abnormality.

### 2.1 The first course of operation: debulking surgery

The patient was clinically diagnosed as having ovarian cancer with ascites, and she was scheduled for surgery on November 25, 2008. Laparotomy revealed a little bloody ascites, 2 massive solid tumors on both ovaries (maximum 8 × 8 cm on the right side, maximum 10 × 8 cm on the left side). Numerous peritoneal implants scattered on the surface of uterus, cystic serosa, rectum and sigmoid colon, and the greater and lesser omentum. The peritoneal cancer index (PCI) was 19. The debulking procedure included hysterectomy, bilateral oophorectomy, and resection of implanted nodules on the peritoneum. But the residual tumor in the Cul-de-sac and in the sigmoid colon mesentery could not be completely removed, as the patient and her family refused preventive colostomy. The completeness of cytoreduction (CC) score was 3. The clinicopathology report was ovarian serous papillary adenocarcinoma (grade II-III).

### 2.2 Interval chemotherapy

Three cycles of adjuvant systemic chemotherapy were delivered on day 9, November 4, 2008 to February 11, 2009, after the debulking surgery, with paclitaxel liposomes at the dose of 175 mg/m^2^, and carboplatin (area under the curve [AUC] −5 mg). The serum CA 125 levels decreased significantly, from 90.8 to 32.9 U/mL. However, reexamination by doppler ultrasonography and CT scan revealed tumor mass at the Cul-de-sac region.

### 2.3 The second course of operation: CRS + HIPEC

After 3 cycles of systemic chemotherapy, the patient was reevaluated by a multidisciplinary team of surgical oncologists specialized in peritoneal carcinomatosis and gynecological oncologists. It was concluded that the patient was fit for another major surgery and there was no absolute surgical contraindication.

On February 25, 2009, the patient received a complete CRS + HIPEC. Intraoperative PCI assessment was conducted immediately after laparotomy following Sugarbaker criteria.^[7]^ The patient had a PCI of 11, with tumor nodules on remnant greater omentum, pelvic peritoneum, and sigmoid colon. In addition, there was moderate adhesion in the lower right quadrant and the pelvic regions. Standardized CRS was performed, including greater omentectomy, complete pelvic peritonectomy with resection of rectosigmoid colon (Fig. 1B), achieving a postoperative CC score of 0. After CRS, an open-technique HIPEC was performed with mitomycin C 30 mg and cisplatin 120 mg at 43 ± 0.5°C for 90 minutes.

The pathology revealed serous papillary EOC with peritoneal metastasis and calcifications. The abdominal tumor has invaded to serosa and muscularis of the sigmoid colon (Fig. 1C).

### 2.4 Consolidation chemotherapy

From February 28, 2009 to August 18, 2009, the patient received 8 cycles of systemic chemotherapy for consolidation purpose. The regimen was paclitaxel liposome at the dose of 175 mg/m^2^, and carboplatin (AUC—5). The regimen was repeated every 3 weeks. No grade 3 or 4 adverse events occurred during the chemotherapy course. The serum CA 125 level remained low during and after chemotherapy (Fig. 1D).

### 2.5 Follow-up

**Figure 2:**
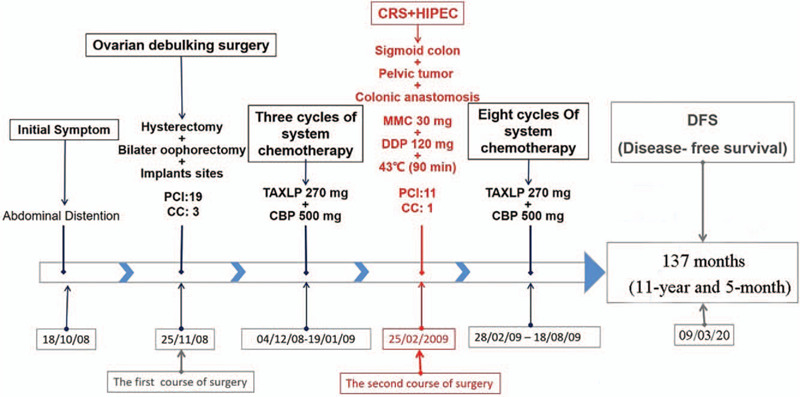
The treatment course of the patient. From the first treatment in November 2008 to the last follow-up in March 2020, the patient has been disease-free for almost 10 years.

The patient has been followed up on a regular basis. By the time of preparation of the manuscript, she remained disease-free survival (DFS) in good condition for >10 years. The treatment course of the patient was summarized in Figure 2. This case report was approved by the ethics committee of Beijing Shijitan Hospital, Capital Medical University and the informed consents were obtained from the patient.

## 3 Discussion

The report presents a case of EOC-PC, successfully treated by integrated multidisciplinary therapy, debulking surgery, interval chemotherapy, CRS+HIPEC, and consolidation chemotherapy. Up to the last follow-up, the patient remains disease free over 10 years. In the background of very dismal clinical outcome for patients with advanced EOC, with 5-year survival <20%,^[8,9]^ this case deserves special attention, as it could offer useful insight into the clinical management of EOC-PC.

The key feature of this successful case is the timely application of the currently best treatment approaches. When the tumor burden was very big, debulking surgery was the only sensible option to achieve a quick tumor burden reduction. The 3 cycles of interval chemotherapy further helped reduce the tumor burden and also render the remnant tumor at very low proliferation status. After the interval chemotherapy, the standard CRS + HIPEC ensured a CC0–1 cytoreduction and total eradication of remnant tumor implants and free cancer cells in the abdomen and pelvis. Furthermore, the 8 cycles of consolidation chemotherapy post CRS + HIPEC was routinely accomplished.

Generally speaking, PC is an inevitable and incurable endpoint of EOC, but innovations in advanced EOC treatment have been going on, with major developments in intraperitoneal chemotherapy.

As early as 1993, Salle et al^[10]^ first reported the application of peritoneal heat perfusion chemotherapy in the treatment of ovarian cancer, and showed certain efficacy and safety. Subsequently, numerous cohorts or case–control studies focused on CRS + HIPEC for primary or recurrent EOC-PC, and obtained encouraging efficacy and acceptable safety compared to traditional treatments. A literature review on these studies were detailed in Table 1.^[11–21]^ Among these studies, 2 prospective randomized control trials (RCTs) deserve special attention.

**Table 1:** Literatures of ovarian cancer treated by CRS + HIPEC in recent 5 years.

					Chemotherapy agents		Median OS, mo		Median DFS, mo		
Author	Year	Country of the publication	Type of the article	Patients (no.)	CRS + HIPEC	System chemotherapy	Primary	Recurrent	Primary	Recurrent	Median follow-up
van Driel et al^[21]^	2018	Netherland	Phase III trial	245	DDP 100 mg/m^2^	TAXOL 175 mg/m^2^ + CBP AUC 6	45.7	—	14.2	—	4.7 y
Mercier et al^[20]^.	2018	France	Retrospective cohort study	210	—	—	69.3	30.3	43.5 mo		
Ceresoli et al^[19]^.	2018	Italy	Retrospective case control	56	DDP 100 mg/m^2^ +TAXOL 175 mg/m^2^	CBP + TAXOL	∗	—	—	—	43 mo
Manzanedo et al^[18]^	2017	Spain	Retrospective study	61	TAXOL 60mg/m^2^ or DDP 100 mg/m^2^+ DOX 15 mg/m^2^	CBP + TAXOL	∗	57	14	17	23 mo
Di Giorgio et al^[16]^	2017	Italy	Retrospective study	511	DDP 75 mg/m^2^, TAXOL or DOX or L-OHP 460 mg/m^2^ or MMC	CBP and TAXOL or DOX liposomal and TPT	54.2	16.6	53.8 mo		
Magge et al^[17]^	2017	United State	Retrospective study	93	DDP 175 mg/m^2^, or MMC 40 mg/m^2^	Null	—	38	—	13.3	—
Sun et al^[15]^	2016	China	Retrospective study	245	DDP 100 mg/m^2^ +TAXOL 100 mg/m^2^ or MMC 20 mg/m^2^ or LBP 50 mg/m^2^	DD 100 mg/m^2^ + TAXOL 100 mg/m^2^/ or DOX 35 mg/m^2^	74	57.5	∗	8.5	45.8 mo
Kocic et al^[14]^	2016	Serbia	Retrospective study	31	DDP 50 mg/m^2^	—	51	19	22 mo		
Spiliotis et al^[13]^	2015	Greece	Phase III trial	120	DDP 100 mg/m^2^ + paclitaxel 175 mg/m^2^ or MMC 15 mg/m^2^ or DOX 35 mg/m^2^	TAXOL 175 mg/kg + CBP AUC 6	—	26.7 (Mean)	—	—	A period of 8 y
Safra et al^[11]^	2014	Israel	Retrospective study	111	DDP 50 mg/m^2^ + DOX 15 mg/m^2^ or TAXOL 60 mg/m^2^+ CBP AUC 4, or MMC 3.3 mg/L/m^2^	DDP or TAXOL or DOX liposomal, TPT, GEM	—	∗	—	15	—
Coccolini et al^[12]^	2014	Korea	Phase II study	54	DDP 100 mg/m^2^, and TAXOL 175 mg/m^2^	—	—	32.9	—	12.5	20 mo

The “∗” indicates the median survival is not reached, “—” indicates the data is not available in the article. CBP = carboplatin, DDP = cisplatin, DFS = disease free survival, DOX = doxorubicin, GEM = gemcitabine, LBP = lobaplatin, L-OHP = oxaliplatin, MMC = mitomycin, OC = ovarian cancer, OS = overall survival, TAXOL = paclitaxel, TPT = topotecan.

Spiliotis et al^[13]^ reported the first phase III RCT in 2015. This trial was conducted for 8 years in Greece. Finally, 120 patients with recurrence EOC-PC (stage IIIc and IV) were included and randomized into CRS + HIPEC group and CRS alone group. With a period of 8 years, OS was significantly longer in the CRS + HIPEC group than CRS alone group (26.7 vs 13.4 months, *P* < .05). Moreover, the 3-year survival rate was 75% in CRS + HIPEC group, much better than 18% in CRS alone group (*P* < .01). They also found that patients with PCI <15 and complete cytoreduction could benefit more from CRS + HIPEC.

van Driel et al^[21]^ reported the first large-sample-size RCT on CRS + HIPEC in primary phase III EOC in 2018. A total of 245 patients with newly diagnosed advanced EOC, fallopian tube carcinoma, and primary peritoneal carcinoma were first treated by 3 cycles of paclitaxel-and-carboplatin adjuvant chemotherapy and then randomized into CRS + HIPEC group and CRS alone group. At a follow-up of 4.7 years, the median recurrence-free survival was 3.5 months longer in CRS + HIPEC group than in the CRS group (14.2 vs 10.7 month). The 3-year recurrence-free survival was 17% in the CRS + HIPEC group and 8% in the CRS only group (*P* = .003). The median OS of CRS + HIPEC group is better than CRS only group (45.7 vs 33.9 months, *P* = .02). And the adverse events of grade 3 or 4 have no difference between the 2 groups.

Based on accumulating high-level clinical evidence, the Peritoneal Surface Oncology Group International (PSOGI) has recommended CRS + HIPEC for selected patients with EOC-PC, although more international collaborations in multicenter RCTs are needed to further validate this integrated treatment package.

This work reported a case of EOC-PC successfully treated by CRS + HIPEC integrated treatment strategy. Combined with literature analysis, this case again provides evidence that complete CRS + HIPEC could be currently the most promising treatment for EOC-PC.

## Author contributions

JZ participated in the conception of the paper and wrote the manuscript. LJM and FBW performed the experiments and analyzed the patient data. YL edited the manuscript. Raw data regarding the patient are managed strictly.

**Conceptualization:** Jue Zhang, Yan Li.

**Data curation:** Liejun Mei, Fubing Wang, Yan Li.

**Methodology:** Yan Li, Jue Zhang.

**Resources:** Liejun Mei, Fubing Wang, Yan Li.

**Supervision:** Yan Li.

**Visualization:** Fubing Wang.

**Writing – original draft:** Jue Zhang.

**Writing – review & editing:** Yan Li.
